# Long Noncoding RNA *DCST1-AS1* Promotes Cell Proliferation and Metastasis in Triple-negative Breast Cancer by Forming a Positive Regulatory Loop with miR-873-5p and MYC

**DOI:** 10.7150/jca.33982

**Published:** 2020-01-01

**Authors:** Li Tang, Yuli Chen, Xun Tang, Da Wei, Xinyu Xu, Feng Yan

**Affiliations:** 1 Department of Clinical Laboratory, Jiangsu Cancer Hospital & Jiangsu Institute of Cancer Research & the Affiliated Cancer Hospital of Nanjing Medical University, Nanjing 210009, P. R. China.; 2 Department of Clinical Laboratory, Nanjing Qixia District Hospital, Nanjing 210000, P. R. China.; 3 Department of Surgery, Jiangsu Cancer Hospital & Jiangsu Institute of Cancer Research & the Affiliated Cancer Hospital of Nanjing Medical University, Nanjing 210009, P. R. China.; 4 Department of Pathology, Jiangsu Cancer Hospital & Jiangsu Institute of Cancer Research & the Affiliated Cancer Hospital of Nanjing Medical University, Nanjing 210009, P. R. China.

**Keywords:** long noncoding RNA, MYC, miR-873-5p, IGF2BP1, metastasis.

## Abstract

**Background:** DC-STAMP domain containing 1-antisense 1 (*DCST1-AS1*) is a long noncoding RNA (lncRNA) that is up-regulated in triple-negative breast cancer (TNBC) tissues. Here, we attempt to investigate the oncogenic property of* DCST1-AS1*.

**Methods:** LncRNA microarrays were used to detect differentially expressed lncRNA in cancerous tissues. Fluorescence *in situ* hybridization assay was used to detect the distribution of *DCST1-AS1* in BT-549 and MDA-MB-231 cells. Lentiviral systems, inhibitors, siRNA and overexpression plasmids were used for gain- and loss-of-function experiments. Colony formation assay, wound healing assay, CCK8 assay, transwell assay, and flow cytometry assay were used to study the function of *DCST1-AS1*. Luciferase assay was used to verify the binding of MYC to the promoter region and the binding of miR-873-5p to *DCST1-AS1*. RNA immunoprecipitation assay was used to verify that argonaute 2 binds to both miR-873-5p and *DCST1-AS1*. Western blotting was used to measure changes in protein expression.

**Results:** Consistent with the microarray results, we found that *DCST1-AS1* was up-regulated in both TNBC tissue samples and cell lines. *DCST1-AS1* was positively correlated with distant metastasis and histopathological grades. *DCST1-AS1* is distributed in both nucleus and cytoplasm. Knockdown of *DCST1-AS1* inhibits TNBC cell proliferation and metastasis, while overexpression of *DCST1-AS1* promotes TNBC cell proliferation and metastasis. We confirmed that *DCST1-AS1* expression in TNBC cells is regulated by MYC. Furthermore, we found that *DCST1-AS1* is negatively correlated with miR-873-5p in TNBC tissues and is a direct target gene of miR-873-5p. Argonaute 2 is involved in the binding of *DCST1-AS1* and miR-873-5p and promotes the degradation of *DCST1-AS1*. The interaction of *DCST1-AS1* with miR-873-5p ultimately up-regulated the expression of insulin-like growth factor 2 mRNA binding protein 1 (IGF2BP1), MYC, CD44 and lymphoid enhancer binding factor 1 (LEF1).

**Conclusions:** *DCST1-AS1* is activated by MYC and is degraded by binding to miR-873-5p, thereby upregulating the expression of miR-873-5p downstream proteins IGF2BP1, MYC, LEF1 and CD44. MYC, *DCST1-AS1* and miR-873-5p form a positive regulatory loop to promote TNBC cell proliferation and metastasis.

## Introduction

Triple-negative breast cancer (TNBC) is a heterogeneous disease in which the estrogen receptor (ER), progesterone receptor (PR), and human epidermal growth factor receptor 2 (HER2) are absent. TNBC has the characteristics of early onset and high rates of invasion and distant metastasis, making it a more aggressive subtype of breast cancer 1, 2. Due to the lack of expression of hormone receptors and HER2, endocrine therapy and trastuzumab treatment are ineffective in TNBC. Therefore, it is urgent to elucidate the molecular mechanisms underlying TNBC metastasis and identify available therapeutic targets 3.

Recent studies have shown that long noncoding RNA (lncRNA), with a length of more than 200 nt, has a wide range of regulatory functions and can regulate gene expression at the level of modification, transcription, and post-transcriptional regulation 4-6. LncRNAs can interact with DNA, RNA, or proteins and are widely involved in the regulation of tumor signaling pathways 7, 8. For example, the lncRNA *MAYA* promotes breast cancer cell bone metastasis through the ROR1/HER3-LLGL2-*MAYA*-NSUN6 signaling axis 9. The LncRNA *LINP1* can be used as a scaffold for Ku80 and DNA-PKcs to promote double-strand DNA breakage repair 10. Although emerging evidence has shown the paramount role of lncRNAs in tumor development, only a small portion of them have been well characterized in various carcinomas 11-13. The number of lncRNAs expressed in TNBC, and whether they have important biological functions, remains largely unknown.

DC-STAMP domain containing 1-antisense 1 (*DCST1-AS1*) is an up-regulated lncRNA identified by microarray analysis. The function of *DCST1-AS1* has not been previously reported. In this study, we verified the results of lncRNA microarrays, elucidated the subcellular localization of *DCST1-AS1*, and explored its diagnostic efficiency and clinical significance. We demonstrated that *DCST1-AS1* is activated by MYC and binds directly to miR-873-5p, ultimately upregulating the expression of insulin-like growth factor 2 mRNA binding protein 1 (IGF2BP1) and its downstream protein MYC, CD44, lymphoid enhancer binding factor 1 (LEF1). *DCST1-AS1* forms a positive feedback loop with miR-873-5p and MYC to promote TNBC cell proliferation and metastasis. This study sheds light on the potential utilization of *DCST1-AS1* as a novel therapeutic target for TNBC.

## Materials and Methods

### Microarray assay

Six pairs of matched primary tumor and adjacent normal tissues were selected from the tissue sample bank. The cancer tissues were confirmed by immunohistochemical staining to be negative for ER, PR, and HER2. The Arraystar Human LncRNA Microarray v4.0 was used to detect lncRNA and mRNA in TNBC tissues. The original signal file of the probe was imported into the GeneSpring GX v12.1 software and standardized to obtain lncRNA expression information. Data is available through the Gene Expression Omnibus GSE115275.

### Tissue samples and cell lines

Thirty pairs of primary TNBC and adjacent normal tissues were obtained from the tissue bank of Jiangsu Cancer Hospital. All tissues were from female patients and were diagnosed between 2017 and 2018. Two experts from the Department of Pathology of Jiangsu Cancer Hospital confirmed by immunohistochemistry that all cancer tissue samples included in the study were ER negative, PR negative, and HER negative. The study was approved by the Ethics Committee of Nanjing Medical University. Informed consent was obtained from all patients. Tissues were frozen in liquid nitrogen until RNA extraction. The TNBC cell lines (MDA-MB-453, BT-549, MDA-MB-231, and HCC1937) and mammary epithelial cell line HBL-100 were obtained from the Chinese Academy of Sciences Cell Bank (Shanghai, China) and cultured according to the ATCC protocols. These cell lines were tested before the study by methods of morphology check, growth curve assay, and mycoplasma detection.

### Isolation of RNA and RT-qPCR

Total RNA was extracted from the cells using Trizol reagent (Invitrogen, Carlsbad, CA, USA) according to the manufacturer's protocol and assessed for quality by One-Drop (Eppendorf, Hamburg, Germany). Mature miRNAs were quantified using the Bulge-Loop miRNA RT-qPCR starter kit and the Bulge-Loop miRNA RT-qPCR primer set (RIBOBIO, Guangzhou, China). The 2-fold Ct method was used to calculate the fold change in gene expression. Primer sequences were shown in Table 1.

### Fluorescence *in situ* hybridization assay

*DCST1-AS1* RNA probes were labeled with Cy3; BT-549 and MDA-MB-231 cells were fixed and incubated with RNA probes. All manipulations were performed according to the instructions of the FISH kit (Gene Pharma, Shanghai, China). After hybridization, nuclear staining was performed using DAPI staining and the intracellular distribution of *DCST1-AS1* was observed with a fluorescence microscope.

### Interference and overexpression assay

The lentiviral interference vector of *DCST1-AS1* and the negative control were synthesized and packaged by Genechem (Shanghai, China). The lentiviral overexpression vector of *DCST1-AS1* and the negative control were synthesized and packaged by GenePharma. The original titers of all infectious viruses were > 10^8^ TU/ml. Gradient dilution was used to determine the multiplicity of infection, puromycin (10μg/ml) was used to screen stably transfected cell lines, and RT-qPCR was used to detect the knockdown or overexpression efficiency. MYC overexpression vector and interference fragment were purchased from GenePharma.

### CCK8 assay

The cells were plated in 96-well culture plates (3 × 10^3^ cells per well). At intervals of 24 hours, 10 μl of CCK8 (Dojindo, Kyushu, Japan) was added to each well and incubated for 2 hours, and the absorbance was read at 450 nm. Five replicate wells were set at each time point and the experiment was repeated at least 3 times.

### Colony formation assay

The cells were seeded into a 6-well plate at a gradient density of 800 cells per dish and gently rotated to uniformly disperse the cells. When macroscopic colonies appeared, the supernatant was discarded and the cells were washed twice with PBS. Cells were then fixed with 2 ml of 4% paraformaldehyde for 15 minutes, stained with 1% crystal violet for 20 minutes, then washed with running water.

### Wound healing assay

A sterile 200 μl pipette tip was used to create a wound on a monolayer of cells and the floating cells were washed away with PBS. Microscopic examination of cell migration near the wound was performed regularly. Each experiment was repeated at least 3 times.

### Flow cytometry assay

The cell cycle was analyzed using PI Staining Kit (KeyGEN, Nanjing, China). Apoptosis was analyzed using Annexin-V APC/7-AAD Double Staining Kit (KeyGEN). The stained cells were then analyzed using a BD LSRFortessa^TM^ flow cytometer (BD Biosciences, Franklin Lake, NJ, USA). Each experiment was repeated at least 3 times.

### Transwell assay

Transwell chambers (MilliporeSigma, Burlington, MA, USA) and BD BioCoat Matrigel Invasion Chambers (Franklin Lake, NJ, USA) were used for cell migration and invasion assays. After incubation, the upper chamber residual cells were removed with cotton-tipped swabs, and the cells that passed through the membrane were fixed with 4% paraformaldehyde and stained with 0.5% crystal violet. For each experiment, the number of migrating tumor cells was counted from 5 randomly selected fields. Each experiment was repeated at least 3 times.

### Luciferase reporter assay

The reporter vectors containing wild type and mutant MYC binding sites were constructed separately. The interaction between MYC and the promoter binding site was confirmed by the increase in relative fluorescence of the reporter gene. The wild type and mutant type of *DCST1-AS1* were cloned into the reporter vector, and the miR-873-5p mimics were co-transfected with the constructed reporter gene vector into 293T cells. The interaction of miR-873-5p with *DCST1-AS1* was confirmed by the decrease in relative fluorescence of the reporter gene. Luciferase activity was measured by Dual-Luciferase Reporter Assay System (Promega, Mannheim, Germany) according to the manufacturer's instructions.

### Western blotting assay

The cells were lysed in RIPA Lysis Buffer (Beyotime, Shanghai, China) containing 1 mM PMSF (Beyotime), and total protein from the cell lysate was extracted. The primary antibodies for MYC (1:1000 dilution; Abcam, ab32072), CD44 (1:1000 dilution; Abcam, ab51037), LEF1 (1:1000 dilution; Abcam, ab53293), IGF2BP1 (1:1000 dilution, Abcam, ab184305), AGO2 (1:1000 dilution, Abcam, ab186733), and GAPDH (1:1000 dilution, Abcam, ab181602), were incubated with the transfer membrane for 2 hours at room temperature. The bands were detected using a SuperSignal West Femto Maximum Sensitivity Substrate Kit (Thermo Scientific, Rockford, USA), the images were acquired using a SYNGENE G: BOX chemiXR5 system (Cambridge, UK), and the results were analyzed using Gel-Pro32 software. GAPDH was used as an internal reference to standardize the relative expression of the proteins.

### RNA immunoprecipitation assay

Sufficient cells were harvested and lysed. RNA immunoprecipitation was performed using the Magna RIP RNA-Binding Protein Immunoprecipitation Kit (Millipore, Billerica, MA, USA) according to the manufacturer's instructions. Immunoprecipitation was performed using an AGO2 antibody (Abcam, ab186733) [or immunoglobulin G (IgG) control]. After washing, the AGO2-bound RNAs were eluted and quantified. RT-qPCR was performed to examine whether miR-873-5p and *DCST1-AS1* were co-immunoprecipitated.

### Statistical analysis

All data presented are representative of at least 3 independent experiments. Wilcoxon signed rank test was used to compare the matched TNBC and adjacent normal samples. The Mann-Whitney *U* test was used to analyze the correlation between *DCST1-AS1* expression levels and clinicopathological features. Student's *t* test and one-way analysis of variance (ANOVA) were used to finish the comparisons. *P* values less than 0.05 were considered statistically significant ( ^#^, *P* < 0.05; *, *P* < 0.01, **; *P* < 0.001). All statistical analyses were performed using SPSS version 22.0 (SPSS, Chicago, IL, USA).

## Results

### *DCST1-AS1* is a novel lncRNA and upregulated in TNBC

**Figure 1:**
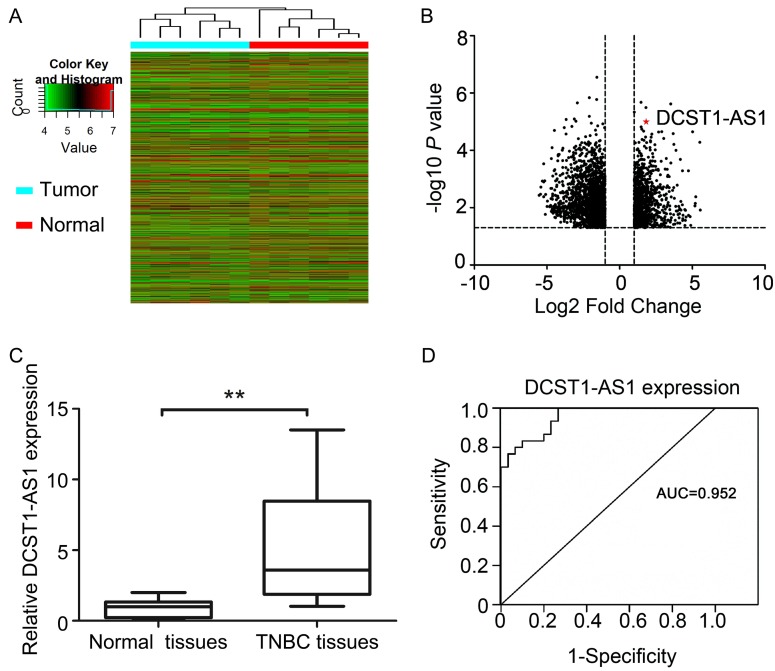
*DCST1-AS1* is up-regulated in TNBC tissues. **(A)** Hierarchical cluster analysis of lncRNAs in 6 pairs of tumor samples and adjacent normal tissue samples. **(B)** Analysis of 6484 lncRNAs that were differentially expressed (≥ 2-fold; P < 0.05) using volcano plots. Of these, 2,464 were up-regulated and 4,020 were down-regulated. **(C)** *DCST1-AS1* expression was analyzed by RT-qPCR in tumor samples and adjacent normal tissues (n = 30). *DCST1-AS1* expression level was normalized to GAPDH. Horizontal lines in the box plots represent the medians; the boxes represent the interquartile range. The significant differences between samples were analyzed using the Wilcoxon signed rank test. **, *P* < 0.0001. **(D)** ROC curve for prediction of TNBC using RT-qPCR based *DCST1-AS1* expression level. The AUC was 0.952, with 95% CI and *P* value indicated,* P* = 0.023.

Hierarchical cluster analysis revealed systematic changes in transcriptional expression levels between cancer tissues and adjacent normal tissues (Figure 1A, B). *DCST1-AS1* is an antisense lncRNA that we screened via microarray detection and bioinformatics analysis. We used RT-qPCR to detect *DCST1-AS1* in 30 pairs of TNBC tissues and adjacent normal tissues, and found that *DCST1-AS1* in cancer tissues was significantly higher than adjacent normal tissues, which was consistent with microarray results (Figure 1C). In addition, receiver operating characteristic (ROC) curve showed that the area under the curve (AUC) was 0.952, [95% confidence interval (CI), 0.906 - 0.998, *P* = 0.023], suggesting that *DCST1-AS1* detection has high sensitivity and specificity (Figure 1D).

### *DCST1-AS1* is associated with tumor size and lymph node metastasis

Patients with pathologically confirmed TNBC were included in this study according to the selection criteria. We grouped these 30 pairs of tissue specimens according to age, menopause, tumor size, histopathological grade, lymph node metastasis, distant metastasis, and histopathological grade. According to the median *DCST1-AS1* detection, the RT-qPCR results of *DCST1-AS1* in TNBC tissues and adjacent normal tissues were divided into a high expression group and a low expression group. The results showed that *DCST1-AS1* was positively correlated with distant metastasis (*P* = 0.024) and histopathological grade (*P* = 0.026) (Table 2).

### Characterization of *DCST1-AS1* sequence and expression pattern

**Figure 2:**
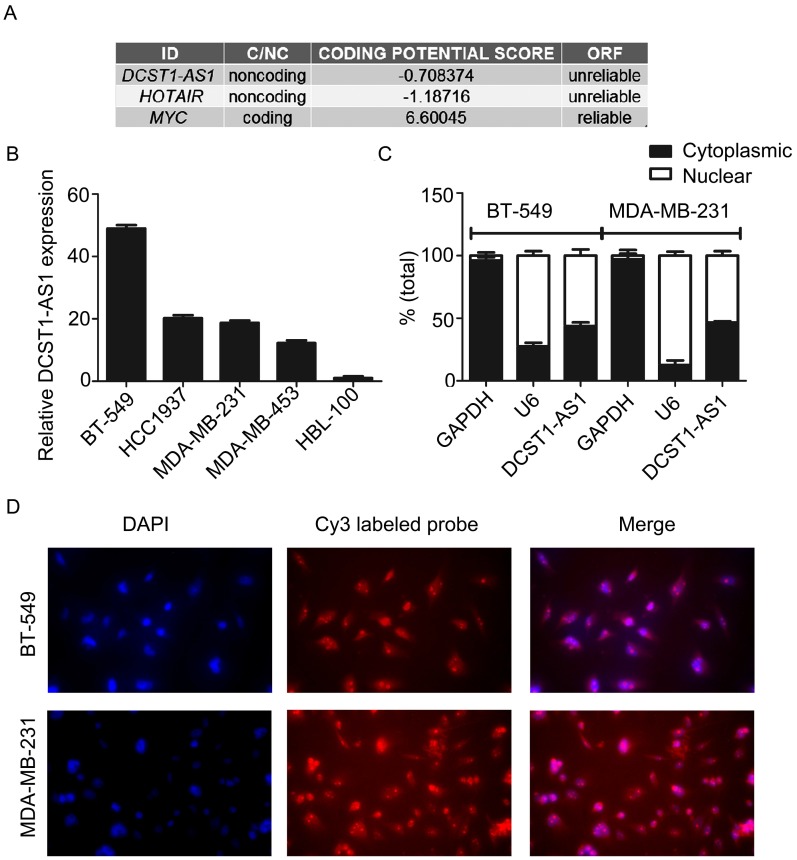
*DCST1-AS1* is a bona fide lncRNA and is distributed both in the nucleus and cytoplasm. **(A)** Analysis of coding capacity of *DCST1-AS1*, *MYC* as a coding gene control and *HOTAIR* as a noncoding gene control. **(B)** RT-qPCR was used to detect the expression of *DCST1-AS1* in BT-549, MDA-MB-231, HCC1937, MDA-MB-453, and HBL-100 cells. The mammary epithelial cell line HBL-100 was used as a reference. Values represent averages and bars represent S.D. of three independent experiments. **(C)** RT-qPCR was used to determine the nuclear/cytoplasmic expression ratio of *DCST1-AS1* in BT-549 and MDA-MB-231 cells. U6 was used as an internal reference for the quality of nuclear RNA extracted, and GAPDH was used as an internal reference for the quality of cytoplasmic RNA extraction. Values represent averages and bars represent S.D. of three independent experiments. **(D)** FISH was used to detect the subcellular localization of *DCST1-AS1*. Nuclei were detected with DAPI staining and *DCST1-AS1* was detected with a Cy3 labeled probe.

The analysis of the Coding Potential Calculator 14 shows that *DCST1-AS1* is a typical noncoding RNA lacking protein coding ability and a complete open reading frame (ORF) (Figure 2A). *DCST1-AS1* was significantly increased in TNBC cell lines (BT-549, HCC1937, MDA-MB-231, and MDA-MB-453) compared to the mammary epithelial cell line HBL-100 (Figure 2B). HBL-100 cells originally derived from a normal source of breast tissue, established by primary cultures of healthy female milk epithelial cells, and whose transformed phenotype is attributed to integrated simian virus 40 (SV40) genetic information. HBL-100 is an immortalized non-tumorigenic mammary epithelial cell line that is negative for estrogen and progesterone receptors and is used as a source of normal human mammary epithelial cells in many studies 15-17. The RT-qPCR showed that *DCST1-AS1* was expressed both in the nucleus and in the cytoplasm, with a slightly higher expression in the nucleus at a distribution ratio of approximately 6:4 (Figure 2C). The subcellular localization of *DCST1-AS1* was detected by fluorescence *in situ* hybridization assay, and results showed that the fluorescence intensity in the nucleus was higher than in the cytoplasm, which was consistent with the nuclear/cytoplasmic RT-qPCR assay (Figure 2D).

### Knockdown of *DCST1-AS1* inhibits TNBC cell proliferation and invasion

**Figure 3:**
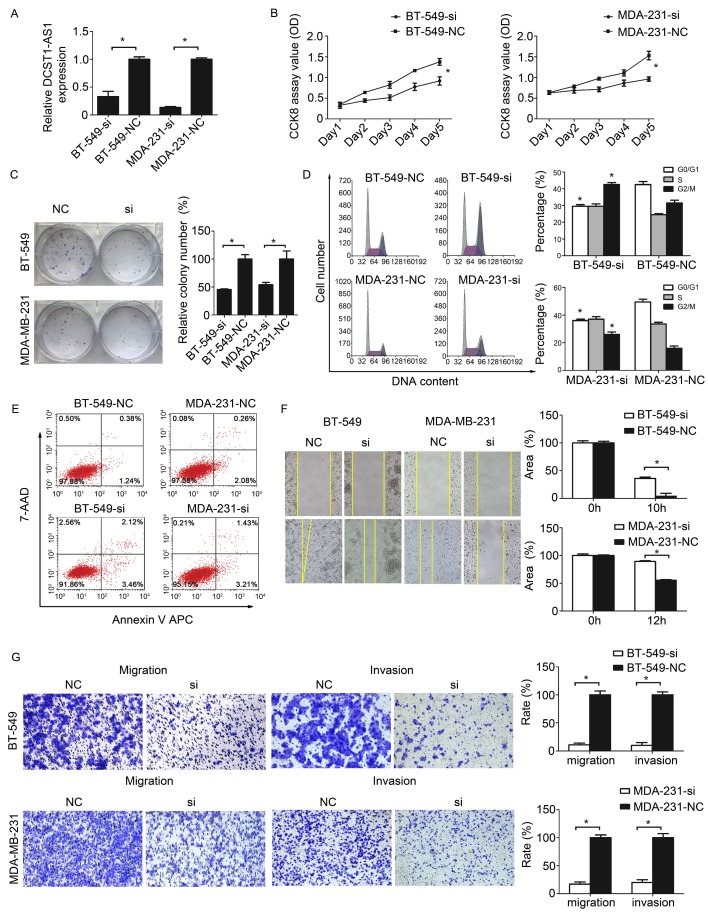
Knockdown of *DCST1-AS1* inhibits proliferation and migration. **(A)** RT-qPCR was used to detect the expression level of *DCST1-AS1* in TNBC cells after lentivirus transfection. *, P < 0.01, compared with the NC group. **(B, C)** CCK8 assay and colony formation assay were used to examine cell proliferation in *DCST1-AS1* knockdown TNBC cells. *, P < 0.01, compared with the NC group. **(D, E)** Flow cytometry was used to detect cell cycle and apoptosis rates of BT-549-si, MDA-231-si cells after lentiviral transfection. *, P < 0.01, compared with the NC group. **(F, G)** Wound-healing assay and transwell assay were used to determine the migration ability of *DCST1-AS1* knockdown TNBC cells. In the migration assay, BT-549 and MDA-MB-231 cells were incubated for 18 hours, whereas in the invasion assay, cells were incubated for 24 hours. *, P < 0.01, compared with the NC group.

To assess the possible role of *DCST1-AS1* in TNBC cells, we obtained two sets of TNBC cell lines stably expressing *DCST1-AS1* siRNA or negative control using lentivirus, labeled BT-549-si, BT-549-NC and MDA-231-si, MDA-231-NC, respectively. Our data indicate that lentiviral interference vectors can effectively knock down endogenous *DCST1-AS1* levels in these cells (Figure 3A). The growth curve detected by CCK8 showed that *DCST1-AS1* knockdown significantly reduced TNBC cell growth (Figure 3B). Cell colony formation assay showed that down-regulation of *DCST1-AS1* significantly inhibited anchor-independent growth of BT-549 and MDA-MB-231 cells, resulting in fewer and smaller colonies (Figure 3C). To explore the underlying mechanism of growth inhibition after *DCST1-AS1* knockdown, we evaluated its effect on cell cycle and apoptosis in BT-549 and MDA-MB-231 cells. Analysis of the cell cycle distribution showed that *DCST1-AS1* down-regulation resulted in a decrease in the G0/G1 phase ratio and an increase in the G2/M ratio in BT-549-si and MDA-231-si, whereas G2/M arrest may contribute to *DCST1-AS1* silence-induced growth inhibition (Figure 3D). Annexin V staining showed that the percentage of early apoptotic cells after *DCST1-AS1* knockdown was not significantly changed relative to the control group (Figure 3E). Wound healing assay showed a reduced migration capacity of the *DCST1-AS1* knockdown cell line (Fig. 3F). Using the transwell system, we found that the invasive ability of TNBC cells was significantly reduced after down-regulation of *DCST1-AS1* (Fig. 3G).

### Overexpression of *DCST1-AS1* promotes TNBC cell proliferation and invasion

**Figure 4:**
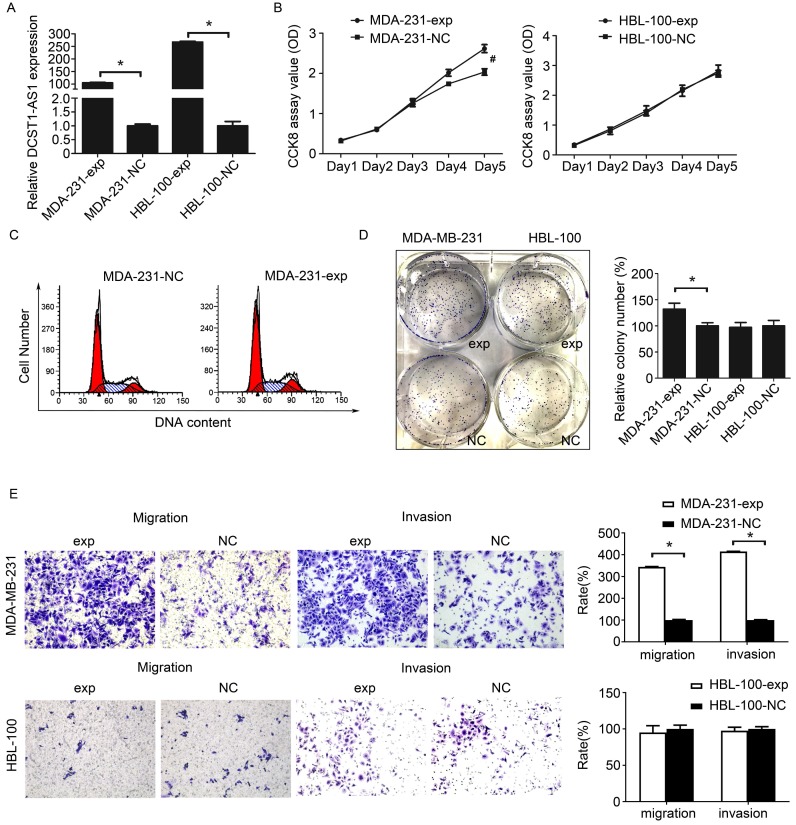
Overexpression of *DCST1-AS1* promotes TNBC cell proliferation and invasion. **(A)** RT-qPCR was used to detect changes in the expression of *DCST1-AS1* in MDA-MB-231 and HBL-100 cells after lentiviral transfection. Transcript levels were normalized to GAPDH. *, P < 0.01, compared with the NC group. **(B)** CCK8 assay was performed to determine the effect of ectopic expression of *DCST1-AS1* on TNBC cell proliferation. #, P < 0.05, compared with the NC group. **(C)** Flow cytometry was used to detect changes in the cell cycle of MDA-MB-231 cells overexpressing *DCST1-AS1*. **(D)** Colony formation assays were used to determine the effect of *DCST1-AS1* overexpression on TNBC cells anchorage-dependent growth. *, P < 0.01, compared with the NC group. **(E)** Transwell assays were used to determine the effect of up-regulation of *DCST1-AS1* on cells invasion. In the migration assay, MDA-MB-231 cells were incubated for 12 hours and HBL-100 cells were incubated for 24 hours. In the invasion assay, MDA-MB-231 cells were incubated for 24 hours and HBL-100 cells were incubated for 48 hours. *, P < 0.01, compared with the NC group.

We still used the lentiviral overexpression system to assess the effect of *DCST1-AS1* overexpression in these hormone receptor negative cells. We selected cell lines with moderate (MDA-MB-231) or very low (HBL-100) endogenous *DCST1-AS1* levels and labeled *DCST1-AS1* overexpressing cell lines as MDA-231-exp and HBL-100-exp, respectively. The expression of *DCST1-AS1* in MDA-MB-231 and HBL-100 cells was significantly increased 96 hours after infection with lentivirus compared to the negative control. The *DCST1-AS1* is up-regulated by 105 times in the MDA-MB-231 cells and up by 266 times in the HBL-100 cells (Figure 4A). The CCK8 assay showed that upregulation of *DCST1-AS1* promoted cell proliferation of MDA-231-exp, but there was no significant change in HBL-100-exp (Figure 4B). Cell cycle analysis indicated that overexpression of *DCST1-AS1* did not alter the distribution of MDA-MB-231 cell cycle (Figure 4C). In the colony formation assay, the number of colonies formed by MDA-231-exp in the overexpressed group was significantly increased after 1 week of culture (Figure 4D). Furthermore, transwell experiments indicated that enhanced expression of *DCST1-AS1* promoted cell invasion of MDA-MB-231 cells (Figure 4E). Although HBL-100 cells overexpressed *DCST1-AS1*, there was no significant change in cell proliferation and invasion ability.

### *DCST1-AS1* is activated by MYC

**Figure 5:**
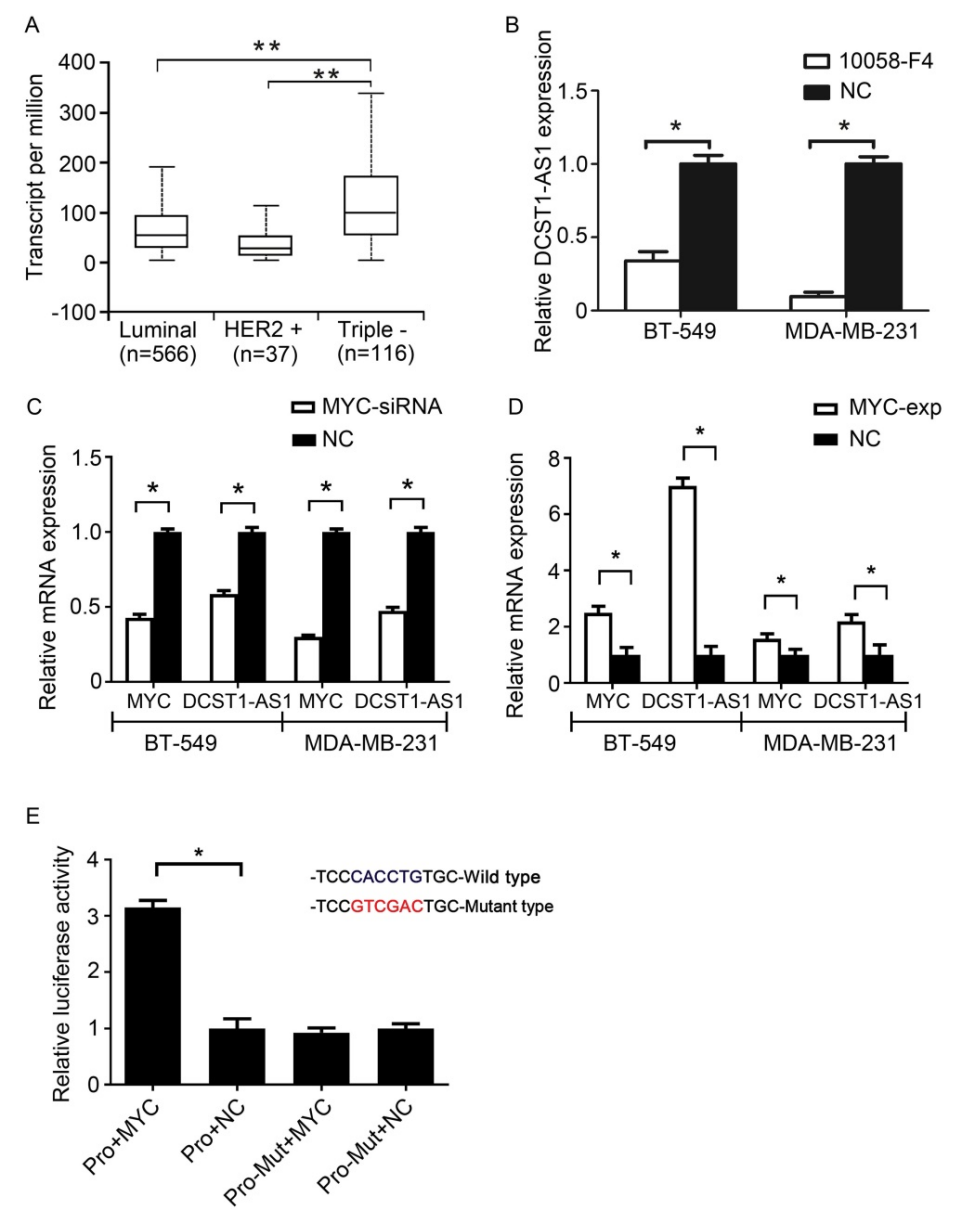
*DCST1-AS1* is activated by MYC. **(A)** Analysis of MYC expression levels in breast cancer subtypes using TCGA data. **, P < 0.0001. **(B, C)** *DCST1-AS1* expression was decreased when MYC siRNA or the MYC-MAX inhibitor 10058-F4 (50 μg/ml) were added to BT-549 and MDA-MB-231 cells. *, P < 0.01, compared with the NC group. **(D)** *DCST1-AS1* expression was up-regulated when MYC-overexpression plasmids were transferred into BT-549 and MDA-MB-231 cells. *, P < 0.01, compared with the NC group. **(E)** Dual luciferase assay confirmed that MYC binds to the *DCST1-AS1* promoter locus. Pro, promoter. *, P < 0.01, compared with the NC group.

By analyzing the Cancer Genome Atlas (TCGA) data, we found high expression of MYC in TNBC (Figure 5A). Recent studies have confirmed that MYC can regulate lncRNAs 18. To confirm whether MYC can regulate *DCST1-AS1* transcription, the MYC-MAX-specific inhibitor 10058-F4 and *MYC* siRNA were used to interfere with MYC expression. RT-qPCR showed inhibition or interference with MYC in BT-549 and MDA-MB-231 cells, also accompanied by a significant decrease in *DCST1-AS1* (Figure 5B, C). In contrast, *DCST1-AS1* in these cells was significantly up-regulated when the MYC overexpression plasmid was transfected (Figure 5D). We predicted the MYC binding site in the 2,000 bp promoter region upstream of the *DCST1-AS1* gene using the JASPAR. The mutated sequence was designed, and the dual luciferase assay was used to detect the binding specificity of the transcription factor and the promoter site. We found that the binding of MYC to the wild type site increased the fluorescence intensity by 3.15-fold, confirming that MYC regulates *DCST1-AS1* transcription (Figure 5E).

### *DCST1-AS1* is direct target of miR-873-5p

**Figure 6:**
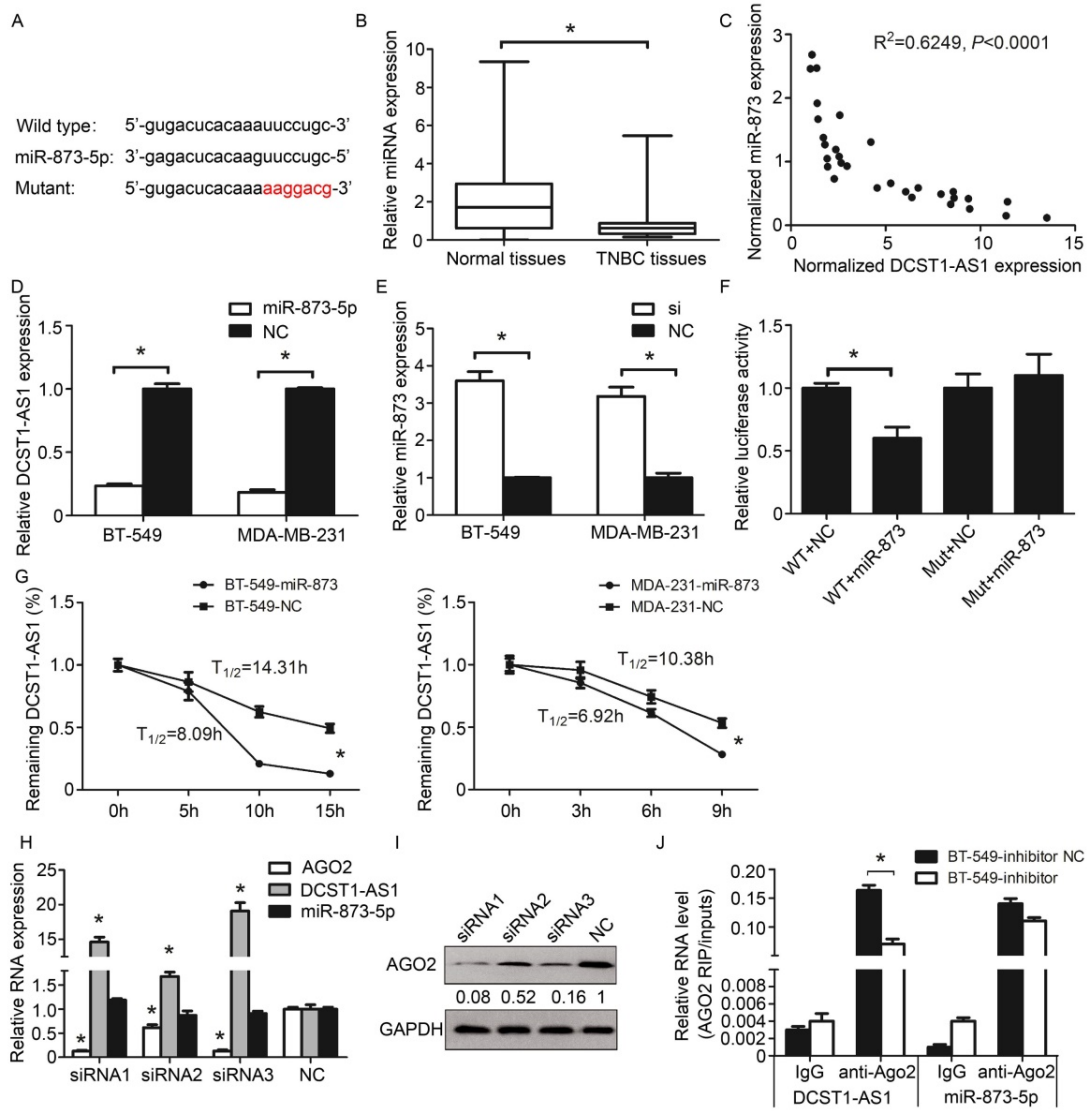
*DCST1-AS1* is direct target of miR-873-5p. **(A)** Prediction of binding sites for miR-873-5p and *DCST1-AS1*. **(B)** Expression of miR-873 in 30 pairs of TNBC tissues and adjacent normal tissues. miR-873-5p expression levels were normalized to U6. The significant differences between samples were analyzed using the Wilcoxon signed rank test. *, P < 0.01. **(C)** The inverse relationship between the expression of *DCST1-AS1* and miR-873-5p. Expression was normalized and correlation was analyzed by Pearson correlation coefficient, R2 = 0.6249, P < 0.0001 **(D)** RT-qPCR analysis showed that *DCST1-AS1* expression was decreased in BT-549 and MDA-MB-231cells transfected with miR-873-5p mimics. *, P < 0.01. **(E)** qPCR analysis showed that miR-873-5p expression was up-regulated by interference with *DCST1-AS1* in BT-549 and MDA-MB-231 cells. *, P < 0.01. **(F)** Dual luciferase assay confirmed that miR-873-5p binds to the predicted binding site of *DCST1-AS1*. WT, wild type. Mut, mutant. *, P < 0.01. **(G)** mRNA stability analysis: The half-life of *DCST1-AS1* in BT-549 cells was shortened by 6.22 hours and the half-life of *DCST1-AS1* in MDA-MB-231 cells was shortened by 3.46 hours; 18S rRNA was used as an internal reference. *, P < 0.01.** (H)** The relative expression levels of miR-873-5p and *DCST1-AS1* in BT-549 cells after interference with AGO2 were detected by RT-qPCR. GAPDH was used as an internal reference for *DCST1-AS1*, and U6 was used as an internal reference of miR-873-5p. *, P < 0.01. **(I)** Western blotting was used to detect IGF2BP1 protein levels in BT-549 cells after AGO2 interference. **(J)** The amount of *DCST1-AS1* and miR-873-5p bound to anti-AGO2 or IgG was measured by RT-qPCR in the presence of a miR-873-5p inhibitor or a negative control. *, P < 0.01.

We predicted that miR-873-5p could bind to *DCST1-AS1* using miRBase (Figure 6A). RT-qPCR assay confirmed that miR-873-5p was down-regulated in TNBC and negatively correlated with *DCST1-AS1* (Figure 6B, C). Overexpression of miR-873-5p in BT-549 and MD-MB-231 cells significantly down-regulated *DCST1-AS1*, whereas knockdown of *DCST1-AS1* in these cells up-regulated miR-873-5p (Figure 6D, E). We designed the mutant sequence based on the "seed region" of miR-873-5p and confirmed that miR-873-5p binds to *DCST1-AS1* through the predicted site by dual luciferase assay (Figure 6F).

MicroRNAs are known to bind in an argonaute 2(AGO2)-dependent manner and cause RNA translational inhibition and/or degradation 19. To understand how miR-873-5p acts on *DCST1-AS1*, we performed mRNA stability experiments. We treated the TNBC cells transfected with miR-873-5p using the RNase inhibitor actinomycin D, and then detected the remaining *DCST1-AS1* in the cells using RT-qPCR. Our results showed that the half-life of *DCST1-AS1* was significantly shortened (Figure 6G), indicating that miR-873-5p affects the stability of *DCST1-AS1* at the post-transcriptional level. Subsequently, we designed the siRNA fragment to knock down AGO2 and found that *DCST1-AS1* was up-regulated by about 19-fold, indicating that AGO2 is involved in the degradation of *DCST1-AS1* (Figure 6H, I). To confirm this inference, we treated BT-549 cells with a miR-873-5p inhibitor or a negative control for RNA immunoprecipitation. We detected *DCST1-AS1* and miR-873-5p in both AGO2 antibody immunoprecipitates, and *DCST1-AS1* was significantly reduced in the miR-873-5p inhibitor group (Figure 6J).

### *DCST1-AS1* regulates the downstream proteins of miR-873-5p

**Figure 7:**
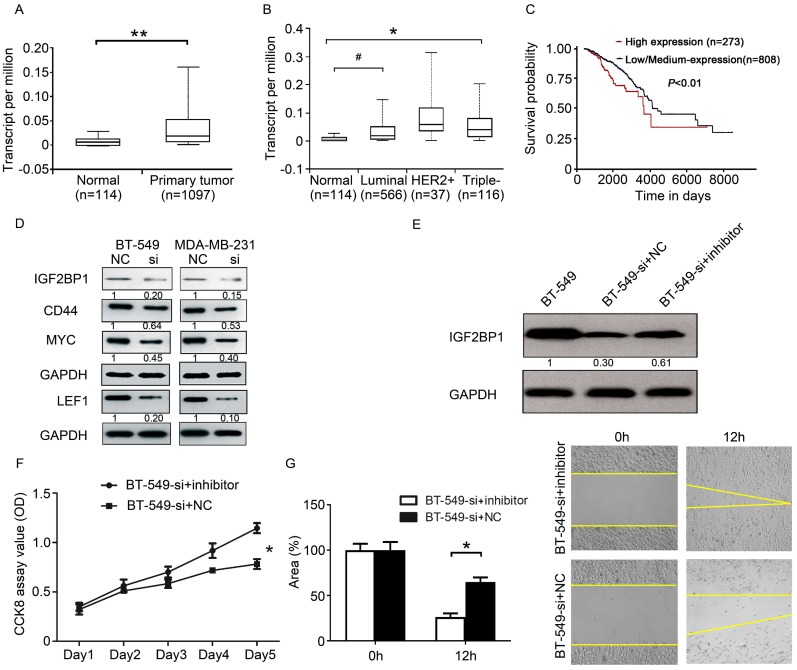
*DCST1-AS1* regulates the downstream proteins of miR-873-5p. **(A)** The expression level of IGF2BP1 in BRCA was analyzed by TCGA data. **, P < 0.0001. **(B)** TCGA data analysis showed that IGF2BP1 expressed higher levels in TNBC and luminal subtypes than normal controls, with significant differences. #, P < 0.05, *, P < 0.01. **(C)** The relationship between the expression of IGF2BP1 and the prognosis of BRCA patients was analyzed by TCGA data. P = 0.0054. **(D)** Western blotting showed a decrease in the expression of IGF2BP1, MYC, LEF1 and CD44 in *DCST1-AS1* interfering cells. **(E)** The miR-873-5p inhibitor was transfected into BT-549-si cells, and the expression of IGF2BP1 protein was measured by Western blotting. **(F)** The miR-873-5p inhibitor was transfected into BT-549-si cells and cell proliferation was measured by CCK8 assay. *, P < 0.01. **(G)** The miR-873-5p inhibitor was transfected into BT-549-si cells and cell migration was measured by wound healing assay. *, P < 0.01.

Since *DCST1-AS1* can affect the expression of miR-873-5p in TNBC cells, can it affect the downstream target genes of miR-873-5p? We searched the literature to find that IGF2BP1 is a direct target gene of miR-873-5p 20. Further analysis of TCGA data, we found that IGF2BP1 is highly expressed in breast cancer (Figure 7A, B). Patients with high IGF2BP1 expression in breast cancer have a worse prognosis (Figure 7C). In the above studies we have confirmed that *DCST1-AS1* is a direct target of miR-873-5p. MYC, CD44 and LEF1 are known downstream proteins of IGF2BP1 and are involved in the regulation of cell proliferation and metastasis. Using Western blotting experiments, we found that knockdown of *DCST1-AS1* effectively down-regulated IGF2BP1, MYC, CD44 and LEF1 protein levels in TNBC cells (Figure 7D). Our study found that *DCST1-AS1* can adsorb miR-873-5p, knockdown of *DCST1-AS1* can increase miR-873-5p in BT-549 cells, thereby reducing the expression of the pro-cancerous factor IGF2BP1. To validate our findings, we designed a series of rescue experiments in which miR-873-5p inhibitors were transfected into BT-549-si cells, and we found that IGF2BP1 expression was up-regulated (Figure 7E). CCK8 detection and wound healing experiments confirmed partial recovery of cell proliferation and migration (Figure 7F, G).

## Discussion

Metastasis is the leading cause of death in solid tumor patients and is considered to be the last step in progressive breast cancer 21. Patients with TNBC have a lower survival rate due to the high invasiveness, metastasis, and rapid recurrence of the tumor 3. The lack of a new treatment for TNBC reflects, in part, a lack of full understanding of its development and progress.

The identification and characterization of cancer-associated lncRNAs is a critical step toward a thorough understanding of cancer biology and subsequent diagnosis and treatment 22, 23. Studies have shown that lncRNAs can regulate many important cancer phenotypes and can also be used as diagnostic or prognostic markers 24-26. We found that *DCST1-AS1* is an oncogenic lncRNA and its expression level is associated with disease progression. Gain- and loss-of-function approaches confirmed that *DCST1-AS1* is involved in regulating cell proliferation and metastasis. To date, there have been no reports on the biological function of *DCST1-AS1*; therefore, this research is innovative.

MYC is a central regulator of tumorigenesis and affects a variety of cellular biological processes 27, 28. It is worth noting that MYC is disproportionately up-regulated in TNBC, and its specific functions are not fully understood. Our results indicate that *DCST1-AS1* is consistent with MYC expression and that MYC can promote *DCST1-AS1* transcription.

It is well known that microRNAs can interact with mRNA by sequence complementarity to induce inhibition of protein translation levels. Recent studies have shown that microRNAs can also bind to noncoding RNAs and play regulatory roles 29, 30. miR-873-5p is a microRNA that is down-regulated in colorectal cancer, gastric cancer, ovarian cancer and breast cancer, and acts as a negative regulator of tumor proliferation and metastasis 31, 32. We found that miR-873-5p is negatively correlated with *DCST1-AS1* in TNBC tissues, and that miR-873-5p binds directly to *DCST1-AS1*. microRNA mainly forms RNA-induced silencing complex (RISC) to negatively regulate target genes, and AGO2 is the core protein that exhibits enzyme cleavage activity 33. We found that *DCST1-AS1* and miR-873-5p bind to the same RISC, and miR-873-5p exerted an mRNA cleavage function at the post-transcriptional level.

IGF2BP1 plays an important role in embryogenesis and carcinogenesis. As a post-transcriptional regulator, IGF2BP1 regulates the expression of some mRNAs required to control tumor cell proliferation and invasion, and is associated with poor overall survival and metastasis in cancer 34. Therefore, IGF2BP1 is considered to be a potential therapeutic target for oncogenes and cancer 35, 36. IGF2BP1 can bind to the unstable determinant in the ORF coding region and prevents the cleavage of *MYC* mRNA by endonucleases and microRNAs 37. IGF2BP1 can increase the stability of *CD44* mRNA and promotes cell adhesion and invadopodia formation in cancer cells 38. IGF2BP1 can prevent *LEF1* mRNA degradation, leading to the up-regulation of LEF1 expression 39. LEF1 is an important transcription factor involved in the activation of the Wnt signaling pathway, and facilitates the synthesis of mesenchymal fibronectin and promotes epithelial-mesenchymal transition in breast cancer 40. We found that the interaction between *DCST1-AS1* and miR-873-5p dilutes the regulation of IGF2BP1 expression by miR-873-5p, upregulating the expression of MYC, CD44 and LEF1, which play important roles in cell proliferation and metastasis. Here, we were pleasantly surprised to find a positive feedback regulation: MYC promotes *DCST1-AS1* transcription, and* DCST1-AS1* increases MYC translation.

We believe that the conclusions of this study are not all the functions and mechanisms of *DCST1-AS1*. In future studies, we will validate the function of *DCST1-AS1* using *in vivo* experiments and screen for potential cellular signaling cascades regulated by *DCST1-AS1* using high-throughput sequencing.

## Conclusions

In summary, we confirmed that *DCST1-AS1* is an oncogenic lncRNA and its expression level is positively correlated with TNBC progression. *DCST1-AS1* is activated by MYC and is degraded by binding to miR-873-5p. *DCST1-AS1* forms a positive regulatory loop with miR-873-5p and MYC to promote TNBC cell proliferation and metastasis. Our research on *DCST1-AS1* will help broaden the understanding of the complex regulatory mechanisms among key molecules in cancer cells and provides a new perspective on the molecular mechanisms of TNBC cell proliferation and metastasis.

## Figures and Tables

**Table 1:** The primer sequences used for RT-qPCR.

Gene name	Forward primer sequences (5'-3')	Reverse primer sequences (5'-3')
*DCST1-AS1*	CCACTCACCAGCTTCTTC	CTTCTGCTATGTCTCACCC
MYC	ACAGCGTCTGCTCCACC	CCTCATCTTCTTGTTCCTCC
AGO2	ATTTCAAGGACAGGCACAA	AAATTCACGGACGTATGGA
GAPDH	ATGGGTGTGAACCATGAGAA	GTGCTAAGCAGTTGGTGGTG

**Table 2:** Relationship between *DCST1-AS1* expression and pathological features in TNBC patients.

Parameter	Number	Relative expression of *DCST1-AS1*		
		High	Low	*P* value
Age (years)				0.204
≤ 50	17	9	8	
> 50	13	4	9	
Menopause				0.963
Yes	8	3	5	
No	22	10	12	
Tumor size				0.267
≤2cm	17	8	9	
>2cm	13	6	7	
LN metastasis				0.563
yes	9	4	5	
no	21	10	11	
Distant metastasis				0.024^#^
yes	10	5	5	
no	20	7	13	
Grade				0.026^#^
G1	7	3	4	
G2-3	23	13	10	

^#^*P* < 0.05. LN, lymph node.

## Abbreviations

TNBC: triple-negative breast cancer
ER: estrogen receptor
PR: progesterone receptor
HER2: human epidermal growth factor receptor 2
lncRNA: long noncoding RNA
*DCST1-AS1*: DC-STAMP domain containing 1-antisense 1
IGF2BP1: insulin-like growth factor 2 mRNA-binding protein 1
AGO2: argonaute 2
LEF1: lymphoid enhancer-binding factor 1
RISC: RNA-induced silencing complex
EMT: epithelial-mesenchymal transition
